# Wireless actuation of micromechanical resonators

**DOI:** 10.1038/micronano.2016.36

**Published:** 2016-08-15

**Authors:** Farrukh Mateen, Carsten Maedler, Shyamsunder Erramilli, Pritiraj Mohanty

**Affiliations:** 1 Department of Mechanical and Aerospace Engineering, Boston University, 110 Cummington Street, Boston, MA 02215, USA; 2 Department of Physics, Boston University, 590 Commonwealth Avenue, Boston, MA 02215, USA

**Keywords:** biomedical implant, MEMS, micromechanical, nanomechanical, wireless actuation, wireless power transfer

## Abstract

The wireless transfer of power is of fundamental and technical interest, with applications ranging from the remote operation of consumer electronics and implanted biomedical devices and sensors to the actuation of devices for which hard-wired power sources are neither desirable nor practical. In particular, biomedical devices that are implanted in the body or brain require small-footprint power receiving elements for wireless charging, which can be accomplished by micromechanical resonators. Moreover, for fundamental experiments, the ultralow-power wireless operation of micromechanical resonators in the microwave range can enable the performance of low-temperature studies of mechanical systems in the quantum regime, where the heat carried by the electrical wires in standard actuation techniques is detrimental to maintaining the resonator in a quantum state. Here we demonstrate the successful actuation of micron-sized silicon-based piezoelectric resonators with resonance frequencies ranging from 36 to 120 MHz at power levels of nanowatts and distances of ~3 feet, including comprehensive polarization, distance and power dependence measurements. Our unprecedented demonstration of the wireless actuation of micromechanical resonators via electric-field coupling down to nanowatt levels may enable a multitude of applications that require the wireless control of sensors and actuators based on micromechanical resonators, which was inaccessible until now.

## Introduction

Wireless energy transfer^1^ consists of energy transfer from any appropriate source to an energy-consuming device^2,3^ and implanted biomedical devices and sensors^4–8^ without the use of physical conductors or solid connecting wires. Predominantly, wireless energy transfer can be realized via either magnetic (B-field) or electric field (E-field) coupling between the source and receiver. Mechanisms involving the inductive coupling of magnetic fields require the short-range placement of an external (source) and an internal (receiver) coil. Mechanisms involving E-field coupling allow the source and receiver antennas to be proximately located. However, at present, they require the microfabrication of LC circuits and the incorporation of highly efficient radio frequency rectifying circuits at the receiver end.

## Materials and methods

**Figure 1:**
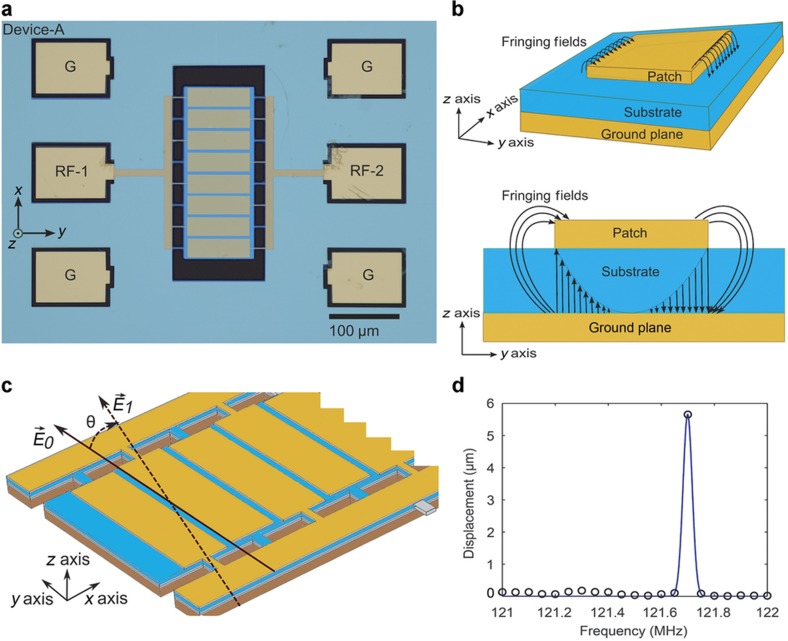
(**a**) Micrograph showing Device A used for the wireless actuation experiments. The four gold tabs marked ‘G’ are the ground terminals, whereas those marked RF-1 and RF-2 may interchangeably be used to apply and measure the input and output signals of the resonator, respectively. The plate-type piezoelectric element of the resonator measures 245 by 100 μm. Eight interdigitated electrodes are overlaid on this element with four connected to the RF-1 tab though thin connects on one side, whereas the remaining four are connected similarly to the RF-2 tab on the opposite side. (**b**) Schematic diagram depicting the working of the patch antenna in both 2D and 3D views. The antenna consists of two parallel conductors (the patch and the ground plane) separated by a suitable dielectric. Fringing fields at the ends of the patch allow the antenna to radiate and receive linearly polarized electromagnetic waves, with the highest directivity in the *z* axis (perpendicular to the patch surface). (**c**) A COMSOL multiphysics model similar to the dimensions and layering details of Device A was developed to show wireless actuation of the resonator. A *y*-polarized E-field is normal incident upon the resonator (solid black arrow). The (golden) interdigitated electrodes sit atop the piezoelectric layer followed by the ground plane, a layer of silicon oxide and silicon. The model is suspended at the center of a cuboid box of air (not shown) serving as the air domain. (**d**) The resulting displacement versus frequency curve from the COMSOL simulation shows that the piezoelectric resonator model (conforming to Device A) resonates at 121.7 MHz under wireless actuation. 3D, three dimensional; 2D, two dimensional.

The piezoelectric effect is manifested as two accompanying inverse and direct effects. The former effect results in a mechanical strain upon the application of an electric field across the material, whereas the latter results in an electric charge polarization in a material upon the application of a strain. The inverse and direct effects are, in turn, used to excite and measure the response of a piezoelectric resonator, respectively. Here we demonstrate wireless actuation of silicon-based micromechanical resonators using conventional piezoelectric resonators. The optical micrograph in Figure 1a shows the top view of Device A, which is one of the two piezoelectric resonators (Devices A and B) used for our demonstration. The devices were fabricated using standard microlithography and surface micromachining. The rectangular resonator is realized by suspending a bottom base layer of structural silicon with a subsequent layer of piezoelectric material (aluminum nitride—AlN) sandwiched between two gold layers, which form the top-patterned interdigitated transducer (IDT) and ground electrodes of the resonator (for more information, refer to Supplementary Information). Typically, an input alternating current signal is applied across these top (IDT) and bottom electrodes to piezoelectrically actuate the resonator. A typical patch antenna, as shown in the schematic in Figure 1b, consists of two parallel conducting plates separated by a dielectric material. Hence, each of the top IDT and bottom electrodes, along with the piezoelectric material (dielectric in our case) sandwiched between them, simultaneously function as an inherent patch antenna, able to capture energy from an incident electromagnetic (EM) wave, and as actuation (or detection) electrodes for the piezoelectric layer below. Patch antennas^9^ can couple to linearly polarized (in the *y* axis) EM waves due to the fringing fields caused by excess charge accumulation at the edges of the top (IDT electrodes in our case) electrodes (Figure 1b) with respect to the bottom ground electrode.

For a resonator lying flat in the *x*–*y* plane (Figure 1c), with the IDT electrode length (*L*), parallel to the *y* axis, the top patch electrodes receive a maximum electric field from a normally incident electromagnetic wave polarized along the *y* axis. This time-varying electric field produces a similarly varying electric potential between the top patch electrodes and the ground plane below, resulting in a strain on the piezoelectric element (inverse piezoelectric effect) that causes the resonator to actuate wirelessly.

## Results and discussion

We have studied two plate-type piezoelectric resonator devices^10^, Device A and Device B, with resonance frequencies of 121.7 and 36.18 MHz, respectively. It is important to note that each of the patch antennas on the resonator has an individual resonant frequency that is dictated by both the relative permittivity of the dielectric substrate (piezoelectric element) over which they are laid and their respective lengths. Hence, for Device A, in which the length (*L*) of each interdigitated electrode (individual patch antenna) is 87.6 μm, the relative permittivity (*ε*_r_) of the substrate (AlN) is taken to be 9.0, and the resonant frequency is found to be 603.33 GHz (for more information refer to Supplementary Information).

A three-dimensional (3D) piezoelectric resonator model conforming to the dimensions and layering structure of Device A was developed and tested using the COMSOL Multiphysics package. The model was simulated using the piezoelectric and radio frequency physics modules, which revealed a wireless-actuated resonant mode at 121.7 MHz upon application of a linearly polarized E-field (along the *y* axis) propagating normally (in the *z* axis) to the resonator surface in the *x*–*y* plane, as shown in Figure 1c (see Supplementary Information for more details).

The radio frequency physics module in COMSOL uses the finite element analysis method to solve the Maxwell equation with sources of the following form.

(1)∇×(∇×E⇀)−ω2ε0µ0µr(εr−iσωε0)E⇀=0

where $\vec{E}$ is the unknown electric field (V m^−1^) to be solved in three dimensions, *ω* is the angular frequency (rad s^−1^), and *μ*_r_, *ε*_r_, and *σ* are the relative permeability, relative permittivity, and electrical conductivity, respectively, as specified by the properties of the material. In addition, *μ*_0_ and *ε*_0_ are the permeability (H m^−1^) and permittivity (F m^−1^) of free space, respectively.

The piezoelectric physics module uses the E-field calculated throughout the finite simulation domain by the radio frequency module and sweeps the same frequency range (121.4 to 122 MHz, step size of 0.05 MHz) to calculate the displacement of the resonator. The study at each frequency step utilizes the respective E-field solution from the radio frequency physics module to calculate the electric potential at the top patch electrodes, which is then provided as an input to compute the displacement of the resonator. This solves the following stress-charge forms of the coupled piezoelectric equations, which are linear in the low *E*-field and mechanical stress regime.

(2)T=cES+etE

(3)D=eS+ε0εrSE

where *S* is the strain tensor of rank 2, *T* is the stress (N m^−2^) tensor of rank 2, *E* is the electric field (V m^−1^) tensor of rank 1, and *D* is the electric charge density (C m^−2^) tensor of rank 1. The material parameters *c_E_* (tensor of rank 4), *e* (tensor of rank 3), and *ε*_rS_ (tensor of rank 2) correspond to the material stiffness (N m^−2^), coupling properties (C m^−2^), and relative permittivity at constant strain, respectively. In addition, *ε*_0_ is the permittivity of free space (F m^−1^), and *e*^t^ represents the transpose of the tensor *e*. Equation (2) describes the inverse piezoelectric effect, whereas Equation (3) describes the accompanied direct piezoelectric effect. The resulting displacement versus frequency curve (Figure 1d) calculated by the simulation validates resonance of the simulated piezoelectric resonator at 121.7 MHz when actuated wirelessly via E-field coupling.

Both Devices A and B were successively tested in the lab to gather extensive distance and the angular dependence data to verify wireless actuation. Each device was wire-bonded to a printed circuit board (PCB). The PCB was mounted on a rotary stage and fixed vertically with its *z* axis (axis shown in Figure 1c) pointing directly towards a transmitting bi-conical antenna. This transmission antenna produced planar EM waves with a horizontally polarized E-field, parallel to the *y* axis of the resonator. The rotary stage served to controllably sweep the resonator’s in-plane (azimuth) angle theta (*θ*; Figure 1c) from 0 to 330 degrees in 12 steps of 30 degrees each. One complete set of such angular measurements was carried out at each of ten distances between 6 and 36 inches from the fixed transmitting antenna, that is, at 6, 8, 10, 12, 16, 20, 24, 28, 32, and 36 inches. These distances each fall within 1 wavelength (calculated at the resonance frequency) for both devices, which is ~2.5 m for Device A and 8.3 m for Device B.

**Figure 2:**
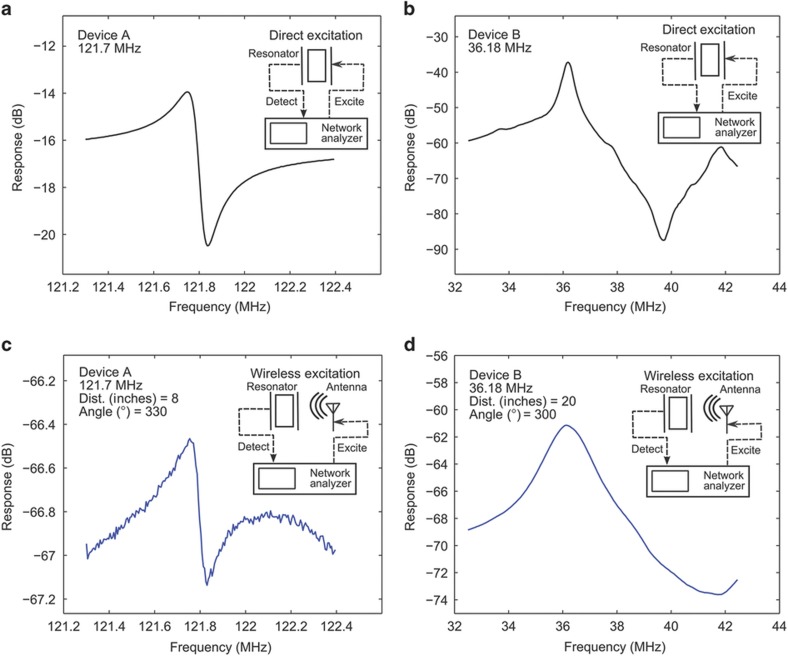
Direct and wireless actuation of both Devices A and B at fixed angles and distances is compared to demonstrate the wireless energy transfer (actuation) of two separate piezoelectric devices. (**a**) The response of Device A excited and measured by a direct excitation via the VNA, as depicted in the inset schematic, is shown. The resonance peak appears at 121.7 MHz. (**b**) The response of device B excited and measured by a vector network analyzer, with a resonance peak at 36.18 MHz. (**c**) Response of Device A excited wirelessly as depicted in the inset schematic. As expected, a similar resonance response is detected for wireless actuation of the resonator. (**d**) Resonance response of Device B excited wirelessly also occurs at exactly the same resonance frequency of 36.18 MHz; however, the magnitude of the response is much smaller. VNA, vector network analyzer.

Altogether, the data sets provided the angular and distance dependence of the wireless actuation of each resonator. We used a vector network analyzer (VNA, Agilent N3383, CA, USA) to record the S21 parameter magnitude and phase at each distance and angle data point. For all data points, the bi-conical antenna connected at port 1 of the VNA was excited at a fixed output power of −10 dBm (0.1 mW) and swept between 121.3 and 122.4 MHz (201-point sweep) for Device A and between 32.5 and 42.5 MHz (201-point sweep) for Device B. The resonator output was recorded at port 2 of the VNA. Figure 2 provides a proof of principle whereby both devices depict wireless resonance responses at exactly the same resonance frequency as when they were tested under direct excitation. Moreover, similar results were obtained when the transmission antenna was excited by VNA and the response of the resonator was recorded on a separate spectrum analyzer. However, this arrangement would not have conveniently yielded the required S21 parameter, which is customary for wireless systems and central to our own data analysis, so the same VNA was used to both excite the source bi-conical antenna and measure the resonator response.

**Figure 3:**
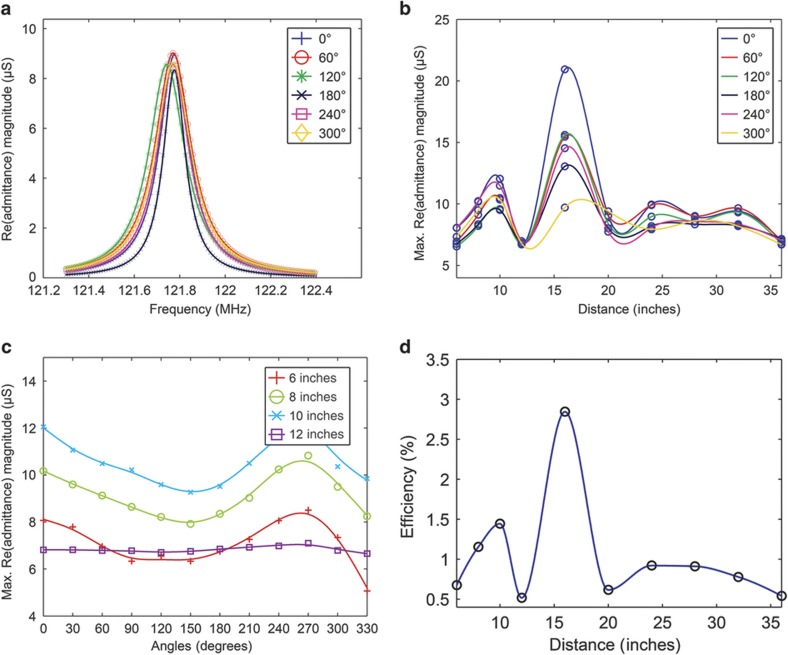
(**a**) The real part (conductance) of the input admittance of an equivalent Butterworth Van Dyke (BVD) circuit, which is a Lorentzian, is plotted for six measured angles of Device A at a 28-inch distance from the fixed bi-conical antenna. The recorded S21 parameter data were used to extract the lumped circuit elements values, which were then used to calculate the real and imaginary parts of the input admittance of the BVD circuit. At each distance, a total of twelve angular measurements were taken, although only six are presented for easier viewing. (**b**) The distance dependence of wireless actuation for Device A is presented. As before, only six (guide-to-eye) plots are presented for easy viewing. A gradual decline is expected with increasing distance; however, owing to near-field effects, multipath interference and reflections less than the ideal response are observed. (**c**) Angular dependence of Device A is presented for 6, 8, 10, and 12 inches. Being linearly polarized, the top-patch electrodes are expected to receive varying amounts of E-field as the in-plane angle theta (*θ*) of the resonator is changed from 0 to 330 degrees. However, it is noteworthy that with increasing distance, this dependence diminishes, possibly due to increased reflections of the waves. As the distance is increased beyond 12 inches, this dependence diminishes further, and only flat plots (not shown) for angular dependence are observed. (**d**) Distance dependence of efficiency for Device A is presented with maximum efficiency of ~3% at 16 inches. The efficiency is the ratio of the output power from the device to the input power irradiating the device at each distance.

The measured S21 parameter raw data represent the ratio of the voltage amplitude at port 2 with respect to that at port 1 of the VNA for a two-port system, consisting of coaxial cables connected to the input and output of a piezoelectric resonator. In particular, the real part of the admittance (*G*_BVD_) given in Equation (4) in terms of the equivalent Butterworth Van Dyke (BVD) model elements is the Lorentzian response of the resonator (see Supplementary Information for the conversion between S21 and *G*_BVD_).

(4)GBVD=RmRm2+(ωLm−1ωCm)2

where *R*_m_, *L*_m_, and *C*_m_ are the equivalent BVD circuit resistance (Ω), inductance (H), and capacitance (F), which represent the mechanical motion of the piezoelectric resonator, and ω is the angular frequency (rad s^−1^). The real part of the admittance (*G*_BVD_) is plotted for each angle for every measured distance. In Figure 3a, we show the plots for only six angles (for easier viewing) for Device A at a distance of 28 inches (chosen at random) from the antenna. The distance dependence of G_BVD_ for Device A (Figure 3b) reveals an unintuitive response, in which it is expected that *G*_BVD_ would gradually decrease with increasing distance, and an anomalous maximum is observed for Device A at 16 inches. A similar anomalous increase for Device B (not shown) is observed at 36 inches. Although both devices do actuate wirelessly, an intuitive understanding of the distance dependence is difficult to form due to the near-field regime effects, reflections, and multipath interference of the linearly polarized systems. To better understand the near-field effects, we have modeled the electromagnetic environment of the lab experimental setup and compared it with that in free space. In free space, both the simulation and experimental data (see Supplementary Information for more details) demonstrate relatively monotonic dependence.

The angular dependence for both Devices A and B also reveals interesting results. Because the transmission (bi-conical) and receiver (patch) antennas are both linearly polarized, the patch receives the maximum of the incident E-field when its in-plane rotation angle theta (*θ*) coincides with the E-field polarization of the bi-conical antenna. As the in-plane angle of each device is varied between 0 and 330 degrees, G_BVD_ is observed to vary non-monotonically. This type of angular dependence is observed for both Devices A (as shown in Figure 3c) and B (not shown). As expected, an amplitude increase is observed at 0 degrees for Device A. However, the amplitude increase in the vicinity of 270 degrees is probably due to the susceptibility of linearly polarized systems to reception under cross-polarization. A similar non-monotonic result is seen for Device B. Furthermore, this behavior is found to be more pronounced for both devices at distances nearer (6, 8, 10, 12 inches) the transmission bi-conical antenna. As the devices are moved farther away from the transmission antenna, beyond 12 inches, amplitude peaks are observed at multiple angles because the device response is overcome by the more predominant near-field and wave reflection effects (Supplementary Information). Although it may seem contrary that the near-field effects become dominant as the distance between the transmission antenna and the device is increased, it may be recalled that all distances at which measurements were carried out were well within a distance of 1 wavelength of the devices; hence, the near-field effects remain dominant.

The efficiencies of both Devices A and B were calculated. Figure 3d displays the distance dependence of efficiency for Device A at a constant in-plane angle *θ* of 0 degrees (see the Supplementary Information for details on efficiency calculation). The incident input power to the device is calculated as the product of the measured power density at each distance by a portable handheld power meter (RF-Explorer 3 G) and the effective area of the top patch antenna array. The output power is calculated from the S21 response of the device measured by the VNA. The ratio of the output to input power reveals the percentage efficiency which for Device A is nearly 3% at 16 inches; for Device B, it is 1% at 32 inches. The distance dependence of efficiency shows that near-field and wave reflection effects do have a role in enhancing or depreciating the received power by the resonator. In Figure 3, it can be observed that Device A has an output signal for nearly all distances and in-plane angle *θ* orientations, and Device B (not shown) displays similar results. However, the efficiency in these first measurements is low, but it can be enhanced by at least an order of magnitude by appropriate design adjustments. For instance, high-directivity antennas are more selective in the direction from which the radiation is received, whereas lower-directivity antennas are susceptible to receiving radiation from multiple directions. This directivity is quantified by the size of the device relative to the wavelength. The patch antennas in our device (~100 μm) are smaller than the wavelengths (~2–8 m) of the radiation, resulting in lower directivity and hence lower efficiency. Appropriate design adjustments to both the resonator and the patch antennas can provide for better directivity and hence increased efficiency.

**Figure 4:**
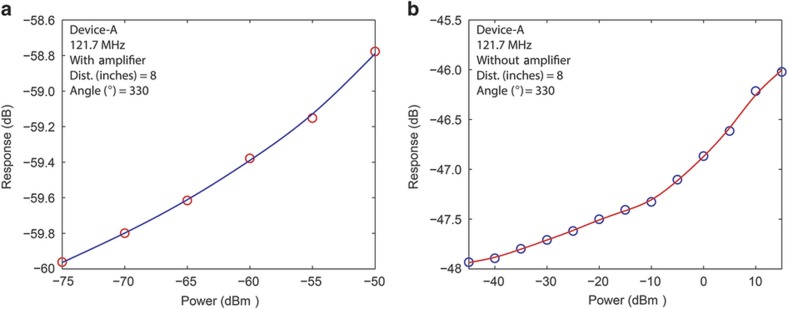
Power sweep plots for Device A. The device is fixed at a certain distance and angle as the power applied to the bi-conical antenna is reduced from 15 dBm to −75 dBm (31.6 mW to 31.6 pW). A 35 dB-gain, low-noise (MITEQ AU-1466) preamplifier is used to amplify the resonator response as the power was reduced below a certain level. (**a**) Plot showing Device A’s response at resonance for a fixed distance and angle of 8 inches and 330 degrees, respectively. The signal was amplified to allow measurements between −50 and −75 dBm (10 nW→31.6 pW). The plot shows the expected gradual decline of the response with decreasing power. Beyond −75 dBm (31.6 pW), the signal-to-noise ratio increases rapidly, increasing the difficulty of obtaining a discernable measurement. (**b**) Plot showing Device A’s resonance response at the same fixed distance and angle for power levels between 15 and −45 dBm (31.6 to 31.6 nW). As before, this non-amplified response is also seen to decrease in magnitude with increasing power.

The studies and plots described to this point, for both Devices A and B, were measured at a fixed EM wave source (bi-conical) antenna output power of −10 dBm (0.1 mW). By fixing the distance and angle of both devices, in turn, we investigate the lowest source antenna power that was still able to generate a discernable resonance response. Device A was fixed at 8 inches and 330 degrees, whereas Device B was fixed at 8 inches and 180 degrees. The source antenna power was swept for both devices between 15 and −75 dBm (31.6 mW to 31.6 pW), whereas the resonance response was observed. A 35-dB gain, low-noise (MITEQ AU-1466) preamplifier was used to amplify the resonator response signals as the power was reduced below −50 dBm for Device A and −45 dBm for Device B. As expected, the guide-to-eye plots for Device A shown in Figure 4 demonstrate that the resonance response amplitude decreases with decreasing source output power. Similar results are obtained for Device B (not shown). Furthermore, a separate similar power sweep experiment (results not shown) for Device A was carried out by fixing it at a distance of 10 inches and at an angle of 330 degrees from the transmission antenna, which resulted in a minimum actuation power of −70 dBm (100 pW).

## Conclusions

To summarize, we demonstrate wireless actuation of micromechanical resonators down to the level of 100 pW of excitation power at a distance >10 inches. Such a low-power wireless excitation technique can pave the way for a host of fundamental experiments that require minimal heating and coupling, as in a quantum system, by the measurement setup. More importantly, these small-footprint, low-power devices, with appropriate design modifications, can be used as wireless power receiving elements in biomedical micro-implants in the brain and the body, enabling a new generation of neuroscience studies that require local targeting with high spatial resolution.

## References

[bib1] Tesla N . Apparatus for transmitting electrical energy. US Patent 1,119,732; 1914.

[bib2] Fernandez JM , Borras JA . Contactless battery charger with wireless control. US Patent 6,184,651; 2001.

[bib3] Kurs A , Karalis A , Moffatt R et al. Wireless power transfer via strongly coupled magnetic resonances. Science 2007; 317: 83–86. 17556549 10.1126/science.1143254

[bib4] Ho JS , Yeha AJ , Neofytoub E et al. Wireless power transfer to deep-tissue micro implants. Proceedings of the National Academy of Sciences of the United States of America 2014; 111: 7974–7979. 24843161 10.1073/pnas.1403002111 PMC4050616

[bib5] Mei H , Irazoqui PP . Miniaturizing wireless implants. Nature Biotechnology 2014; 32: 1008–1010. 10.1038/nbt.3038 25299922

[bib6] Chow EY , Yang C-L , Ouyang Y et al. Wireless powering and the study of RF propagation through ocular tissue for development of implantable sensors. IEEE Transactions on Antennas and Propagation 2011; 59: 2379–2387.

[bib7] Zimmerman MD , Chaimanonart N , Young DJ . In vivo RF powering for advances biological research. 28th Annual International Conference of the IEEE Engineering in Medicine and Biology Society (EMBS 2006); 30 Aug–3 Sep, 2006; New York, NY, USA; 2006: 2506–2509. 10.1109/IEMBS.2006.259571 17945719

[bib8] Kim T-I , McCall JG , Jung YH et al. Injectable, cellular-scale optoelectronics with applications for wireless optogenetics. Science 2013; 340: 211–216. 23580530 10.1126/science.1232437 PMC3769938

[bib9] Constantine A . Antenna Theory: Analysis and Design, 3rd edn. John Wiley & Sons: New York City, NY, USA; 2016.

[bib10] Imboden M , Mohanty P . Dissipation in nanoelectromechanical system. Physics Reports 2014; 534: 89.

