# Supplementary material for: Wireless actuation of micromechanical resonators
Source: Microsyst Nanoeng. 2016 Aug 15;2:16036. doi: 10.1038/micronano.2016.36 (PMC6444740; doi:10.1038/micronano.2016.36)
Supplement: Supplementary Information [file micronano201636-s1.pdf]

Supplementary file

Wireless actuation of micromechanical resonators

Farrukh Mateen[1], Carsten Maedler[2], Shyamsunder Erramilli[2] and Pritiraj Mohanty[2]

Microsystems & Nanoengineering (2016) **2,** 16036; doi:10.1038/micronano.2016.36; Published online: 15 August 2016

1. Figure S1 below shows an optical micrograph of Device B under 20x magnification which is similar in construction to Device A (Figure 1a). The plate-type piezoelectric resonator is overlaid with nine interdigitated top electrodes. Five of them are connected to the RF-1 tab (not shown) on the left side via thin connects while four of the remaining electrodes are connected to the RF-2 tab (not shown) on the right side through similar thin connects.
2. The COMSOL Multiphysics package uses the inhomogeneous wave equation with sources (charges and currents) of the following form, found in most graduate level electromagnetism texts[1], used for solution of the electric field (E-field) in the computational domain:

$$\nabla\times\left(\nabla\times\vec{E}\right)-\omega^2\varepsilon_0\mu_0\mu_r\left(\varepsilon_r-\frac{i\sigma}{\omega\varepsilon_0}\right)\vec{E}=0 \qquad \text{(S1)}$$

Where E is the unknown electric field ($\mathrm{V\,m^{-1}}$) to be solved in 3D, ω is the angular frequency ($\mathrm{rad\,s^{-1}}$), and μ_r, ε_r and σ are the relative permeability, relative permittivity and electrical conductivity respectively, specified by the properties of the material. In addition μ_0 and ε_0 are the permeability ($\mathrm{H\,m^{-1}}$) and permittivity ($\mathrm{F\,m^{-1}}$) of the free space, respectively. The above frequency domain form of the equation assumes that the resultant electric fields will be 'wave-like' and that the power transfer will occur primarily through radiation.
3. The COMSOL[2] coupled piezoelectric resonator Equations (S2) and (S3) in the stress-charge form (so named due to the dependent variables on the LHS of each equation) are of the following form:

$$T=c_E S+e^t E \qquad \text{(S2)}$$

$$D=eS+\varepsilon_0\varepsilon_{rS}E \qquad \text{(S3)}$$

where S is the strain tensor of rank 2, T is the stress ($\mathrm{N\,m^{-2}}$) tensor of rank 2, E is the electric field ($\mathrm{V\,m^{-1}}$) tensor of rank 1,

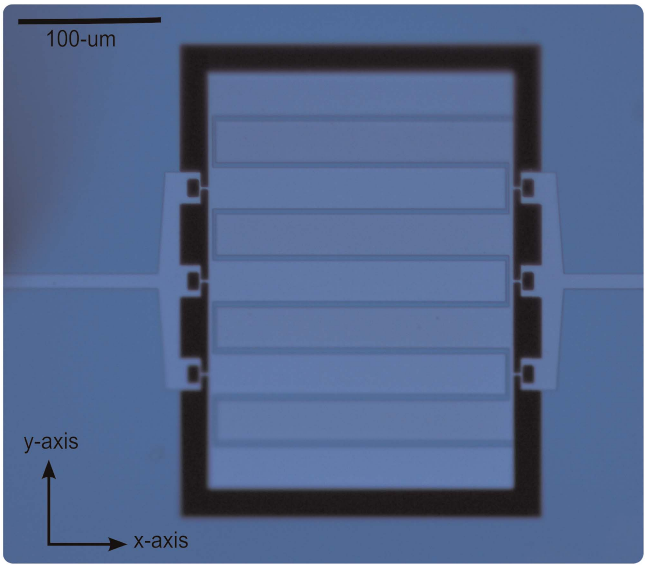

Figure S1 The optical micrograph shows Device B used for the wireless energy transfer experiments in the lab, taken at 20x magnification. It displays a plate-type piezoelectric resonator overlaid with nine interdigitated top electrodes.

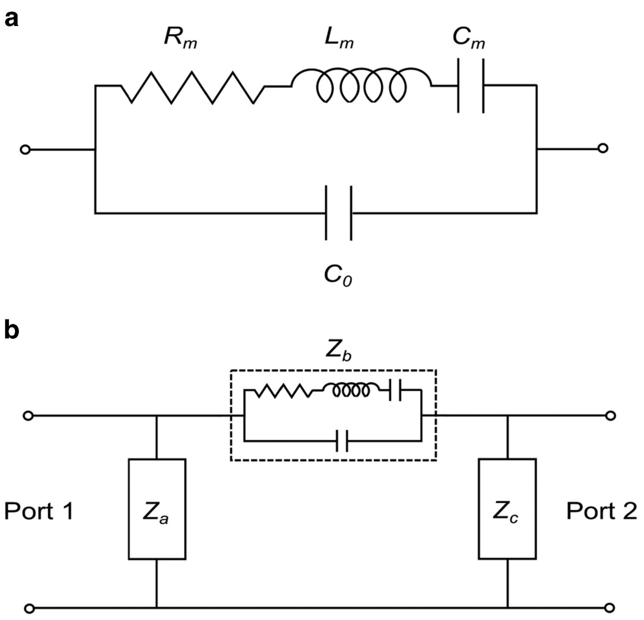

Figure S2 (**a**) The four element Butterworth Van Dyke equivalent model used to model the piezoelectric resonator used in the experiment. It consists of two parallel arms of which the series arm (top) consists of R_m, L_m and C_m which represent the mechanical motion of the resonator while the second arm (below) consists of the capacitance C_0. (**b**) A two-port three-element pi-network is used to model the entire piezoelectric resonator along with connecting coaxial cables. Each element of the network has internal impedance (Z_a, Z_b and Z_c).

[1]Department of Mechanical and Aerospace Engineering, Boston University, 110 Cummington Street, Boston, MA 02215, USA and [2]Department of Physics, Boston University, 590 Commonwealth Avenue, Boston, MA 02215, USA
Correspondence: Pritiraj Mohanty (mohanty@physics.bu.edu)

and D is the electric charge density (C m^{-2}) tensor of rank 1. The material parameters c_E (tensor of rank 4), e (tensor of rank 3) and ε_{rS} (tensor of rank 2) correspond to the material stiffness (N m^{-2}), coupling properties (C m^{-2}) and relative permittivity at constant strain, respectively. In addition, ε_0 is the permittivity of free space (F m^{-1}) and e^t represents the transpose of the tensor e. Equation (S2) describes the indirect piezoelectric effect, while equation (S3) describes the accompanied direct effect piezoelectric effect.

4. The Butterworth Van Dyke[3] (BVD) model is traditionally used to simplify and characterize the piezoelectric resonator and consists of four lumped element components. The model (Figure S2a) consists of two arms in parallel; the first containing Capacitance C_0, which is a purely electrical quantity physically representing the top electrodes and the bottom ground plane, while the second arm contains the resistance R_m, inductance L_m, and capacitance C_m. Qualitatively, R_m, L_m and C_m represent the motional arm and determine the 'series' resonance, where the admittance of the resonator rises to value '$1/R_m$' at a frequency where the series inductance L_m cancels the series capacitance C_m.

The $S21$ data was recorded for a two-port system, consisting of not only a piezoelectric resonator but also a coaxial cabling system connecting the input and output ports of the resonator. To model such a system a two-port, three-element, pi-network was used (Figure S2b). Each of the three elements in the network has discrete internal impedances namely Z_a and Z_c corresponding to the cable connections and Z_b corresponding to the BVD model of the piezoelectric resonator. The measured S-parameter data relating the ratio of output to input voltage, for this two-port pi-network was then converted to Z parameters (Z_{11}, Z_{21}, Z_{12} and Z_{22}) for the same, relating the input and output voltage and currents of the two-port network using conversion formulas given in literature[4], using line impedance Z_0 of 50 Ω. Employing the reciprocity of a passive element network, we have $Z_{21}=Z_{12}$, giving the following Z-parameter equations in terms of internal impedances, Z_a, Z_b and Z_c of the pi-network.

$$Z_{11}=\frac{Z_a(Z_b+Z_c)}{Z_a+Z_b+Z_c}$$

$$Z_{12}=\frac{Z_aZ_c}{Z_a+Z_b+Z_c}$$

$$Z_{22}=\frac{Z_c(Z_a+Z_b)}{Z_a+Z_b+Z_c}$$

The above equations can be inverted to yield explicit expressions for each impedance.

$$Z_a=\frac{Z_{11}Z_{22}-Z_{12}^2}{Z_{22}-Z_{12}}$$

$$Z_b=\frac{Z_{11}Z_{22}-Z_{12}^2}{Z_{12}}$$

$$Z_c=\frac{Z_{11}Z_{22}-Z_{12}^2}{Z_{11}-Z_{12}}$$

All transformations up to this point are exact. However, the only approximation and possible source of error is modeling of the resonator and the connections by a three-element pi-network, while ignoring the more complex parasitic topologies. However, in first order approximations, this model is valid and can provide reasonable results. In particular, the impedance Z_b is of interest as it represents the input impedance of the BVD model circuit. A Matlab algorithm performs these calculations on each measured data set and converts it to the impedance Z_b from which are extracted the series and parallel resonance frequency f_s and f_p along with the corresponding impedance values z_s and z_p at the respective frequencies. These values are used with the following relations to extract the lumped elements of the BVD.

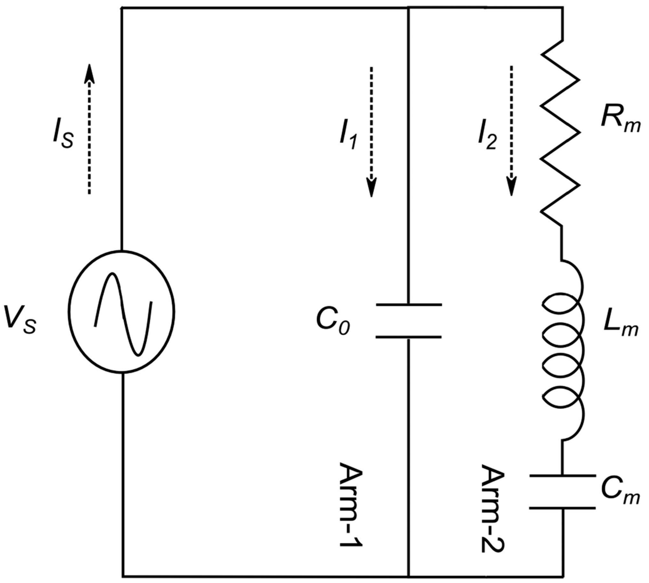

Figure S3 The BVD model under voltage (V_s) excitation results in the total current response I_s flowing in the circuit which is the sum of the currents I_1 and I_2 through each arm as shown. The voltage across each arm is equal to V_s.

$$R_m=z_s$$

$$C_0=\sqrt{\frac{1}{\omega_p^2Rz_p}}$$

$$C_m=C_0\left(\frac{\omega_p^2}{\omega_s^2}-1\right)$$

$$L_m=\frac{1}{C_m\omega_s^2}$$

Here, ω_s and ω_p are the corresponding angular frequencies to both f_s and f_p respectively.

The response of the BVD circuit to a voltage excitation reveals the input admittance of the model, which is derived as follows. Considering a voltage excitation of the form

$$V_s=V_0\exp^{i\omega t}$$

This excitation results in the following current response I_1 and I_2 having same frequency 'ω' as the exciting voltage in each arm, 1 and 2 (Figure S3):

$$I_1=I_{01}\exp^{i\omega t}$$

$$I_2=I_{02}\exp^{i\omega t}$$

For Arm-1 the following Voltage equations results:

$$V_s=\frac{Q_1}{C_0}$$

where Q is the charge and is related to the current by the following relation:

$$Q = \int I dt$$

Hence, the equation for I_1 in terms of the exciting voltage V_s

$$I_1 = V_s i\omega C_0$$

Similarly, the voltage equation for arm-2 becomes

$$V_s = I_2 R_m + \frac{Q_2}{C_m} + L_m \frac{dI_2}{dt}$$

Using the charge-current relation from before and the voltage-current relation for an inductor,

$$V_L = L_m \frac{dI}{dt}$$

results in the following equation for arm-2 current I_2 in terms of exciting voltage V_s

$$I_2 = \frac{V_s}{R_m - \frac{i}{\omega C_m} + i\omega L_m}$$

The total current response of the circuit then becomes

$$I_s = I_1 + I_2$$

$$I_s = V_s i\omega C_0 + \frac{V_s}{R_m - \frac{i}{\omega C_m} + i\omega L_m}$$

$$\frac{I_s}{V_s} = i\omega C_0 + \frac{1}{R_m - \frac{i}{\omega C_m} + i\omega L_m}$$

The LHS of the above equation is the input admittance of the BVD circuit (Y_{BVD}), which is complex, and its real (G_{BVD}) and imaginary (B_{BVD}) parts reveal the amplitude and phase information given by the following equations after simple algebraic manipulations:

$$Y_{BVD} = \left(\frac{R_m}{R_m^2 + \left(\omega L_m - \frac{1}{\omega C_m}\right)^2} \right) + i\left(\omega C_0 - \frac{\left(\omega L_m - \frac{1}{\omega C_m}\right)}{R_m^2 + \left(\omega L_m - \frac{1}{\omega C_m}\right)^2} \right)$$

$$G_{BVD} = \frac{R_m}{R_m^2 + \left(\omega L_m - \frac{1}{\omega C_m}\right)^2}$$

$$B_{BVD} = \omega C_0 - \frac{\left(\omega L_m - \frac{1}{\omega C_m}\right)}{R_m^2 + \left(\omega L_m - \frac{1}{\omega C_m}\right)^2}$$

The earlier calculated values of the series Resistance (R_m), Inductance (L_m) and Capacitance (C_m) from the impedance data Z_b is used to plot the real part of the admittance (G_{BVD}), which is a lorentzian.

5. As shown in the schematic presented in Figure 1b the electric field undergoes fringing along the length of the patch. These fringing fields travel in both the substrate and air hence an effective dielectric constant $\varepsilon_{\mathrm{eff}}$ is used. In addition the same fringing fields causes the patch antenna to appear longer thus an effective Length L_{eff} is introduced. Analytical expressions for calculation of the effective length, dielectric constant and the resonant frequency of the patch are presented below[5].

$$\varepsilon_{\mathrm{reff}} = \frac{\varepsilon_r + 1}{2} + \frac{\varepsilon_r - 1}{2}\left[1 + 12\frac{h}{W}\right]^{-\frac{1}{2}}$$

$$\Delta L = 0.412h \frac{(\varepsilon_{\mathrm{reff}} + 0.3)\left(\frac{W}{h} + 0.264\right)}{(\varepsilon_{\mathrm{reff}} - 0.258)\left(\frac{W}{h} + 0.8\right)}$$

$$L_{\mathrm{eff}} = L + 2\Delta L$$

Where L and W are the length and width of the patch respectively while ε and h are the dielectric constant and height of the piezoelectric material respectively. The resonant frequency of a patch antenna is given by the following equation[5]:

$$f_r = \frac{c}{2L_{\mathrm{eff}}\sqrt{\varepsilon_{\mathrm{reff}}}}$$

where c is the speed of light given approximately as 300 m s^{-2}.

Thus for a patch of L 87-μm, W of 25 μm, piezoelectric substrate height of 2-μm and a dielectric constant of 9.00, all corresponding to one of the patches of Device A the resonant frequency as per the above is given by 603.33 GHz.

The efficiency of Device A is dependent on the efficiency of the top interdigitated array of patch electrodes. The efficiency of this array is given as the ratio of the power the device outputs to the wireless power captured by the device from the transmission bi-conical antenna. The output power from the device can be calculated using the $S21$ parameter from the network analyzer (VNA) data. The input power into Device-A is taken as the power irradiating the resonator due to the transmission bi-conical antenna at each of the 10 measurement distances (6,8,10,12,16,20,24,28,32 and 36 inches). This input power is the product of the power density at each of these distances, as measured by a portable power meter, and the effective area (A_{eff}) of the top interdigitated array of patch antennas. The maximum effective area (A_{eff}) of any antenna is related to its maximum directivity by[5],

$$A_{eff} = \frac{\lambda^2 D_0}{4\pi}$$

Where, lambda (λ) is about 2.5 m corresponding to the irradiating wave at a frequency of 121.7 MHz and D_0 is the directivity of the top interdigitated patch electrode array. This directivity (D_0) is the product of the directivity of a single patch electrode (D_1) and the array factor. Realizing that a patch antenna can be modeled as two radiating slots separated by a length L_{eff} the far-field electric field pattern is then given as[5],

$$E_\phi = +j\frac{k_0 h W E_0 e^{-jk_0 r}}{2\pi r}\left\{\sin\theta \frac{\sin X}{X}\frac{\sin Z}{Z}\right\}$$

$$X = \frac{k_0 h}{2}\sin\theta\cos\phi$$

$$Z = \frac{k_0 W}{2}\cos\theta$$

also $E_r \approx E_\Theta \approx 0$ in the far-field. In addition, for small substrate heights $k_0 h < < 1$ so the $\sin(X)/X$ term becomes = 1, hence we get

$$E_\phi = +j\frac{k_0 h W E_0 e^{-jk_0 r}}{2\pi r}\left\{\sin\theta \frac{\sin Z}{Z}\right\}$$

where

E_ϕ = electric field (V m^{-1})
h = patch substrate height (2e^{-6}m)
W = width of the patch (25e^{-6} m)

f_r = resonance frequency of the patch 603.33 GHz
λ_0 = free space wavelength given by c/f_r (5.00e^{-4} m)
k_0 = free space wave number given by $2pi/\lambda_0$ (12.566e^3 m^{-1})
From the above the radiation intensity U (W/sr) is given by,

$$U = \frac{r^2}{2\eta_0}\left[\left|E_\phi\right|^2\right] = \left(\frac{r^2}{2\eta_0}\right)\left(\frac{k_0 W h E_0}{2\pi r}\right)^2\left\{sin\theta\frac{sinZ}{Z}\right\}^2$$

Where $|E_\Phi|^2 = E_\Phi . E_\Phi^*$. Also η_0 is the impedance of free space about 377 Ω. Considering that the product of the electric field and the height of the substrate gives the voltage across each slot we have, $V_0 = E_0 h$. Power radiated P_{rad} (W) can be calculated via the integration of the radiation intensity U (W/sr), over the solid angles.

$$P_{\text{rad}} = \oiint U d\Omega = \int_0^{2\pi}\int_0^{\pi} U sin\theta d\theta d\phi$$

P_{rad} is given in Watts (W) and the element solid angle $d\Omega = sin\Theta d\Theta d\Phi$, thus integrating the radiation intensity equation over the solid angles, we get P_{rad} as,

$$P_{\text{rad}} = \frac{|V_0|^2}{2\pi\eta_0}\int_0^{\pi}\left(\frac{sin\left(\frac{k_0 W}{2}cos\theta\right)}{cos\theta}\right)^2 sin^2\theta sin\theta d\theta$$

Directivity of an antenna is the ratio of the radiation intensity (U_{max}) in a given direction from the antenna to the radiation intensity averaged over all directions (U_0). The average radiation intensity = $U_0 = (P_{\text{rad}})/4\pi$.

$$D_1 = \frac{U_{\text{max}}}{U_0} = \frac{4\pi U_{\text{max}}}{P_{\text{rad}}}$$

U_{max} = max radiation intensity (W/solid angle)
U_0 = isotropic source radiation intensity (W/solid angle)
P_{rad} = total power radiated (W)
The maximum radiation intensity given as U_{max} (W/sr) as follows,

$$U_{\text{max}} = \left(\frac{r^2}{2\eta_0}\right)\left(\frac{k_0 W h E_0}{2\pi r}\right)^2$$

which reduces to,

$$U_{max} = \frac{|V_0|^2}{2\eta_0\pi^2}\left(\frac{\pi W}{\lambda_0}\right)^2$$

where

$$k_0 = \frac{2\pi}{\lambda_0}$$

Thus the directivity of a single slot using the above becomes,

$$D_1 = \frac{U_{\text{max}}}{U_0} = \frac{4\pi U_{\text{max}}}{P_{\text{rad}}} = \frac{4\pi\frac{|V_0|^2}{2\eta_0\pi^2}\left(\frac{\pi W}{\lambda_0}\right)^2}{\frac{|V_0|^2}{2\pi\eta_0}I},$$

where

$$I = \int_0^{\pi}\left(\frac{sin\left(\frac{k_0 W}{2}cos\theta\right)}{cos\theta}\right)^2 sin^2\theta sin\theta d\theta$$

$$D_1 = \left(\frac{2\pi W}{\lambda_0}\right)^2\frac{1}{I_1}$$

A numerical estimation for the integral I, is given as

$$I_1 = -2 + \cos(X) + XS_i(X) + \frac{sin(X)}{X}$$

Where,
W = width of the patch (25e^{-6} m)
h = height of the substrate (2e^{-6} m)
f_r = resonance frequency of the patch 603.33 GHz
λ_0 = free space wavelength given by c/f (5.00e^{-4} m)
k_0 = free space wave number given by $2pi/\lambda_0$ (12.566e^3)
$X = k_0 W = 0.3$
S_i is the sine integral for which the values of $S_i(X)$ can be found from tables[5]
I_1 for $X = 0.30$ is calculated to be 0.03.
The directivity D_1 for the first slot comes out to be 3.2898 (dimensionless). The directivity of the second slot can be calculated considering that the two slots separated by distance L_{eff} form a linear array of two elements for which the directivity (D_{slots}) is give by[5],

$$D_{\text{slots}} = \frac{2Nd}{\lambda}$$

$N = 2$ (number of elements)
d = distance between the slots L_{eff} (8.8696e^{-5} m).
$\lambda = \lambda_0$ free space wavelength (5e^{-4} m)
$D_{\text{slots}} = 0.7095$
Using $D_1 = 3.2898$ and $D_{\text{slots}} = 0.7095$ the directivity for one patch is given by

$$D_{\text{patch}} = D_1 * D_{\text{slots}}$$

Giving $D_{\text{patch}} = 2.334$ which is the directivity of a single patch antenna for Device A. There are 4 sets of interdigitated patch antennas in Device A (8 patch antennas in total) out of these the signal output is only from one set of 4 patch antennas. Hence the product of the directivity of a single patch (2.334) and the directivity of the linear array of 4 patch antennas each separated form the other by a distance d of 37.86μm gives an array directivity D_{array} of 0.6057, using $N = 4$, and $\lambda = \lambda_0$ in,

$$D_{\text{array}} = \frac{2Nd}{\lambda}$$

This gives the total directivity, D_0 as

$$D_0 = D_{\text{patch}} * D_{\text{array}} = 2.334 * 0.6057 = 1.4138$$

Which gives the total directivity of our patch antenna system as D_0 equal to 1.4138 (dimensionless). This total directivity can be used now to calculate the effective area (A_{eff}) of the patch antenna as given by the expression before. Once the effective area is known the input power to Device A can be calculated and thus the efficiency as described earlier.

6. Figure S4 below shows the schematic fabrication scheme of the piezoelectric resonators.

The piezoelectric resonators are fabricated in a top-down scheme. The wafer used consists of the following layers:

- 1 μm aluminum nitride (AlN) as the device layer.
- 100 nm of gold (Au) as the ground—electrode used for piezoelectric actuation.
- 5 μm of Single crystal silicon (SCS) used as the sacrificial layer.
- 1 μm of silicon oxide
- 800 μm of poly silicon as the handle layer.

The wafer layers are presented in Figure S4a. PMMA is spun at 1600 rpm for 40 seconds. The device pattern is transferred to the PMMA via E-beam lithography (EBL) using an s.e.m. with a beam blanker (Figure S4b). After the pattern is developed 250 nm thick layer of Gold (Au) is deposited via e-beam evaporator (Figure S4c). Following this deposition and subsequent lift-off, the AlN is etched using a Reactive Ion Etching (RIE) process (Figure S4d). The RIE uses a sulfur hexafluoride (SF6) and argon mixture in a ratio of 25/30 sccm respectively, at 300 W and 35 mTorr pressure. This recipe etches away the micron thick AlN layer in about 40 min (Figure S4e). The top and ground electrodes are patterned by applying PMMA again as

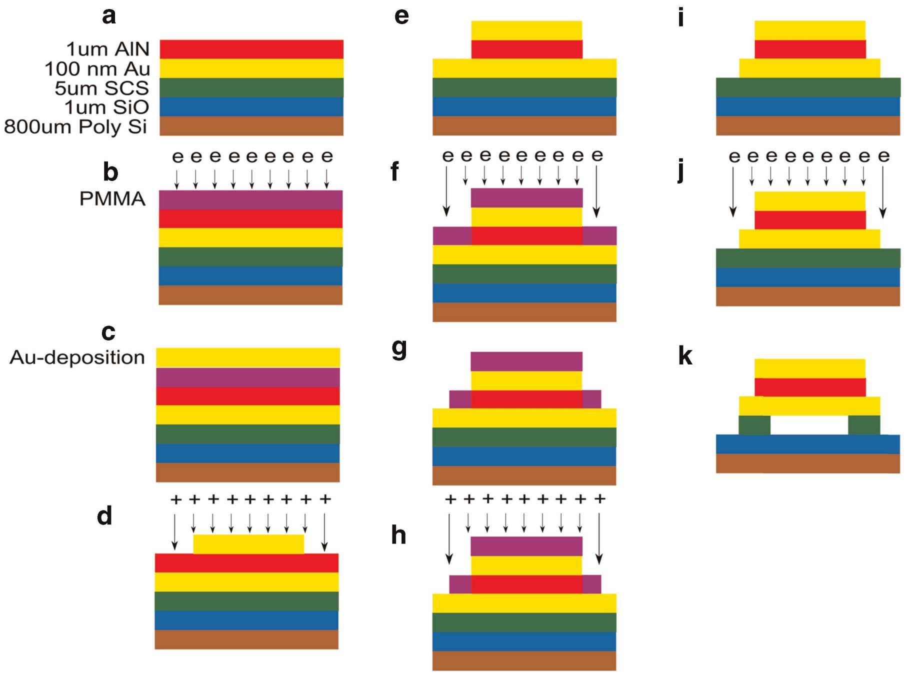

Figure S4 Shows the process flow diagram of the piezoelectric resonator fabrication. (**a**) The wafer used consists of multiple layers which are operated upon in a top-down fabrication method to reveal the resonators. (**b**) Wafer coated with PMMA is operated upon by e-beam lithography. (**c**) Gold deposition. (**d**) Lift-off creates the desired pattern with metal mask. Anisotropic etch of AlN with RIE. (**e**) Piezoelectric resonator etched. (**f**) PMMA is spun and top and bottom electrode patterns are transferred via e-beam lithography. (**g**) Pattern is developed. (**h**) RIE anisotropic etch of top and bottom electrodes. (**i**) Top and bottom electrodes are etches. (**j**) With metal mask isotropic etch, RIE, of sacrificial layer. (**k**) Resonator is suspended.

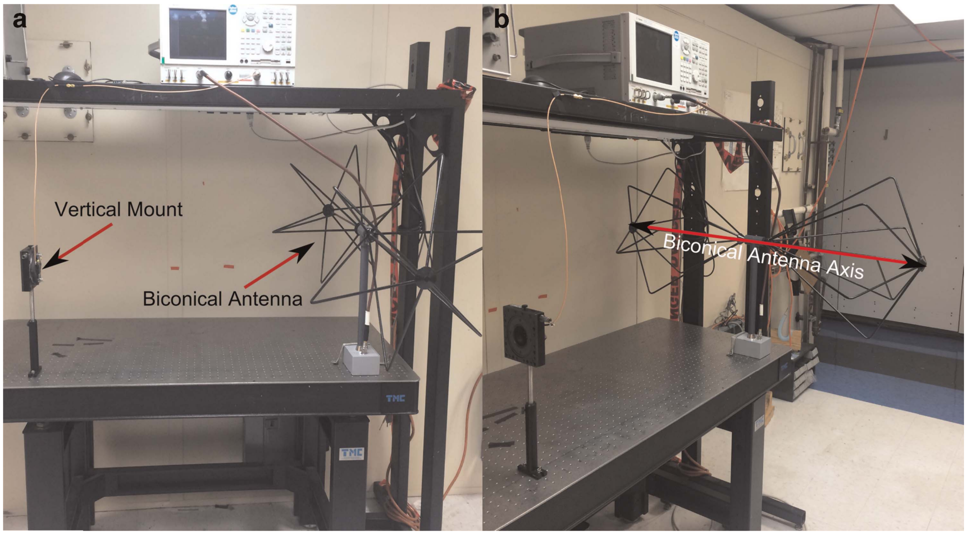

Figure S5 The experimental setup in the lab. (**a**) Side view of the setup showing the vertical mount on which the resonators wire bonded to the PCB are affixed. The mount is moved in the straight line on the optical table from the biconnical antenna. (**b**) Another view of the setup showing the axis of the biconnical antenna. In both views the Vector Network Analyzer is placed in the rack above the optical table.

before and transferring the pattern via EBL (Figure S4f). The resulting pattern (Figure S4g) is etched using the RIE for approximately 3 min using the AlN recipe as before (Figure S4h), revealing the top and ground electrodes (Figure S4i). Once the sacrificial layer is exposed (Single crystal silicon) the RIE is employed again to release the resonator structure (Figure S4j). A low power SF6 plasma gives an almost isotropic etch of the sacrificial layer (Figure S4k). The power and pressure are set to 150 W and 200 mTorr, respectively. The etch rate is found to be rather non-linear, which was slower for the first minute or so. Only direct observation under an s.e.m. can ensure the complete suspension of the resonator structure.

7. It is well known that linearly polarized systems are susceptible to multi-path interference and reflections. We assert that the

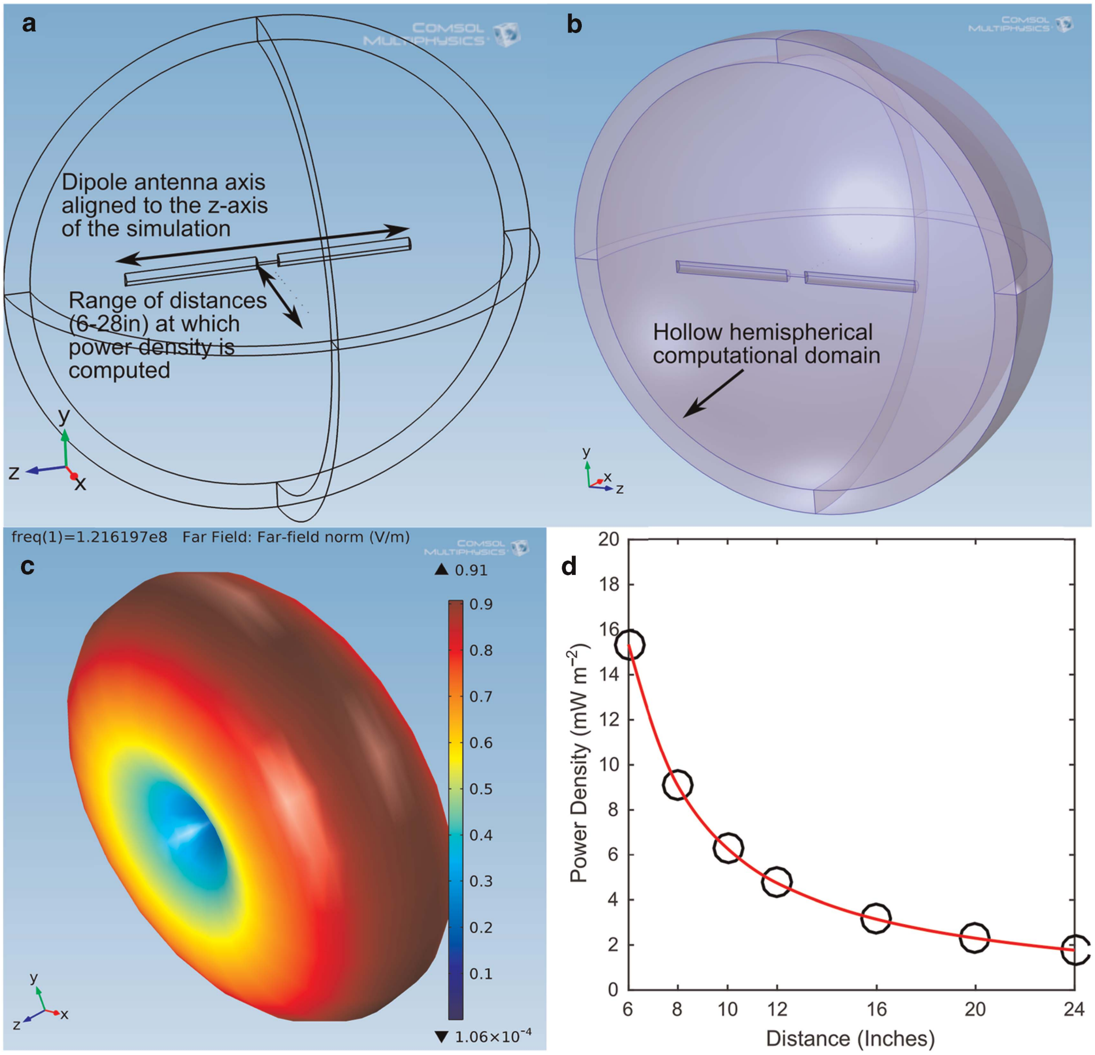

Figure S6 (**a**) Shows the hemispherical computational domain and the model dipole antenna. Power density measurements are made at each of the distances between 6–28 inches. (**b**) Another view of the hemispherical domain. (**c**) The resultant dough-nut shaped radiation pattern of a dipole antenna. (**d**) The power density vs. distance plot showing a gradual decline.

non-intuitive response observed in our data (refer Figure 3b in the manuscript) is due to interference and reflections caused by the presence of reflective surfaces such as optical table, walls, reflective surfaces, data measurement equipment etc. To provide credence to this assertion we present COMSOL simulations, which measure power density at specified intervals (6–28 inches) for both an ideal and non-ideal situation, as, explained below. We also provide results of an experiment carried out outside the lab where reflective surfaces are far removed.

Two views of our in-lab experimental setup are presented in Figure S5. The micron sized piezoelectric resonators are wire-bonded to a PCB that is in turn affixed on the vertical mount facing the biconnical antenna directly as shown. All un-necessary equipment's were removed from the vicinity of the experimental setup. Nearby walls and racks however are fixed and cannot be removed.

COMSOL simulations were made to calculate the power density ($W\,m^{-2}$) at each of the 6, 8, 10, 12, 16, 20, 24 and 28 inch distances from a fixed antenna source for both an ideal scenario (without the presence of any lab equipment's, walls, optical tables etc.) and a non-ideal scenario which took into account the presence of the immovable and essential lab data equipment, with the following assumptions for simplification:

a. A dipole antenna is used in the simulations since it is operationally similar to a biconnical antenna.
b. The spherical computational domain for both simulations around the dipole antenna is halved via a symmetric plane, which serves to reduce computational memory requirements and time.

For both simulations and subsequent results discussed the dipole antenna axis (as shown in Figure S5b) is aligned with the z-axis of the simulation, as per the axis-orientation markers. Figures S6a and b shows views of the hemispherical computational domain where power density ($W\,m^{-2}$) measurements are carried out from 6–28 inches. Figure S6c shows the ideal radiation pattern of a dipole antenna, which is a dough-nut shaped ring, revolved about the axis of the antenna (z-axis of the simulation). Figure S6d shows the plot of the power density vs. distance (inches) and depicts an intuitive gradual decline response as the receiver (resonator) is moved away from the source.

Figure S7 shows the simulation and results related to the non-ideal simulation. Similar calculations for power density

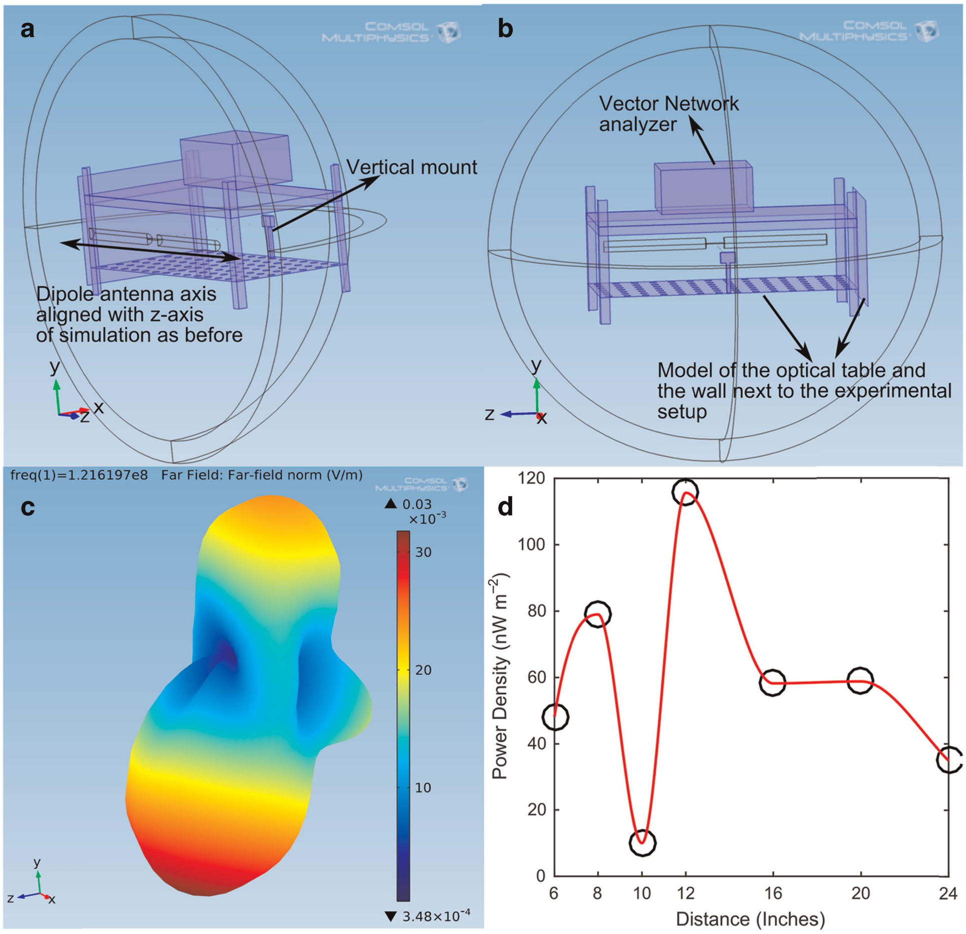

Figure S7 (**a**) Shows the hemispherical computational domain containing the dipole antenna and models of the optical table, adjacent wall, top rack and VNA. Power density measurements are made at each of the distances between 6–28 inches. (**b**) Another view of the same hemispherical domain. (**c**) The resultant distorted radiation pattern of the dipole antenna due to reflections and multi-path interference. (**d**) The power density vs. distance plot showing un-intuitive response.

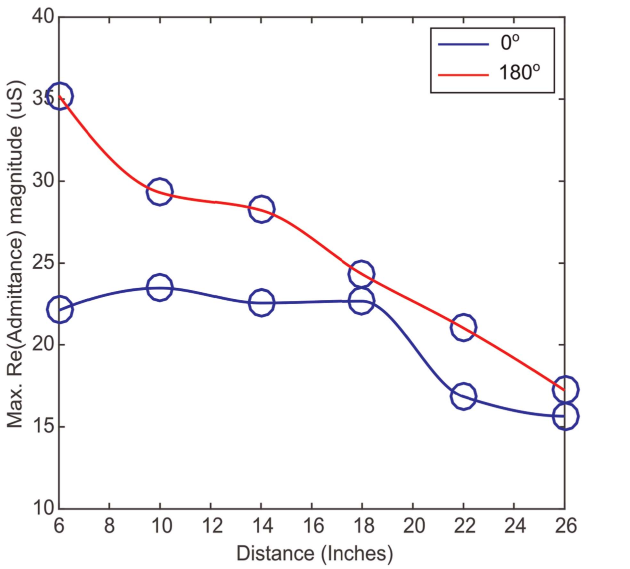

Figure S8 Shows the open-air experiment results for 0 and 180 degree orientations. An approximate monotonic decline can be seen.

($W\,m^{-2}$) as before were carried out at each of the distances between 6–28 inches. A rough model of the optical table and rack, as seen in Figures S5a and b above, is introduced in the simulation to observe the effect of reflective surfaces. Figures S7a and b show two views of the simulation. Due to the presence of the reflecting surfaces the ideal doughnut shaped radiation pattern of Figure S6c is distorted now as seen in Figures S7c. This causes a non-intuitive response in the Power density vs. distance plot in Figures S7d.

While the simulations performed are not to validate the results of the experiments but to provide an understanding that the presence of reflecting surfaces cause the non-intuitive responses observed in our data. However it may be observed that the shape of the response of Figures S7d is similar to that of the experimental response of GBVD vs. Distance of Figure 3b in the manuscript.

Furthermore, we present (Figure S8) the results of the experiments carried out, outside the lab (open-air) where the nearest reflecting wall was at least 8 to 10 wavelengths away from the resonator and source antenna setup with the exception of only the measurements equipment's which had to be placed nearby. The results of GBVD vs. distance for 0 and 180-degree polarizations depict an approximate monotonic and intuitive decline. Thus providing credence to the assertion that the non-intuitive data observed is due to the presence of the reflecting surfaces near our experimental setup.

REFERENCES

1 Anupam G. Classical Electromagnetism in a Nutshell. Princeton University Press: Princeton, New Jersey, USA. 2012.
2 James R. Piezoelectric materials: Understanding the standards, COMSOL. Available at: https://www.comsol.com/blogs/piezoelectric-materials-understanding-standards/ (accessed on Oct 02, 2014).
3 Christian C, Andreas K. MEMS based circuits and systems for wireless communication. Springer Science & Business Media: Berlin, Heidelberg, Germany. 2012; Chapter 1.
4 David M. Microwave Network Analysis, Microwave Engineering, 4th edn. John Wiley & Sons: New York City, USA. 2009.
5 Constantine A. Antenna Theory: Analysis and Design, 3rd edn. John Wiley & Sons: New York City, USA. 2016.
